# Environmental Factors Associated With Nitrogen Fixation Prediction in Soybean

**DOI:** 10.3389/fpls.2021.675410

**Published:** 2021-06-15

**Authors:** André Froes de Borja Reis, Luiz Moro Rosso, Larry C. Purcell, Seth Naeve, Shaun N. Casteel, Péter Kovács, Sotirios Archontoulis, Dan Davidson, Ignacio A. Ciampitti

**Affiliations:** ^1^Department of Agronomy, Kansas State University, Manhattan, KS, United States; ^2^Department of Crop, Soil, and Environmental Sciences, University of Arkansas, Fayetteville, AR, United States; ^3^Department of Agronomy and Plant Genetics, University of Minnesota, Saint Paul, MN, United States; ^4^Department of Agronomy, Purdue University, West Lafayette, IN, United States; ^5^Department of Agronomy, Horticulture, and Plant Science, South Dakota State University, Brookings, SD, United States; ^6^Department of Agronomy, Iowa State University, Ames, IA, United States; ^7^Davidson Agronomics and Consulting, Omaha, NE, United States

**Keywords:** symbiotic nitrogen fixation, relative abundance of ureides, elastic net, LASSO, ridge

## Abstract

Biological nitrogen (N)-fixation is the most important source of N for soybean [*Glycine max* (L.) Merr.], with considerable implications for sustainable intensification. Therefore, this study aimed to investigate the relevance of environmental factors driving N-fixation and to develop predictive models defining the role of N-fixation for improved productivity and increased seed protein concentration. Using the elastic net regularization of multiple linear regression, we analyzed 40 environmental factors related to weather, soil, and crop management. We selected the most important factors associated with the relative abundance of ureides (RAU) as an indicator of the fraction of N derived from N-fixation. The most relevant RAU predictors were N fertilization, atmospheric vapor pressure deficit (VPD) and precipitation during early reproductive growth (R1–R4 stages), sowing date, drought stress during seed filling (R5–R6), soil cation exchange capacity (CEC), and soil sulfate concentration before sowing. Soybean N-fixation ranged from 60 to 98% across locations and years (*n* = 95). The predictive model for RAU showed relative mean square error (RRMSE) of 4.5% and an R^2^ value of 0.69, estimated *via* cross-validation. In addition, we built similar predictive models of yield and seed protein to assess the association of RAU and these plant traits. The variable RAU was selected as a covariable for the models predicting yield and seed protein, but with a small magnitude relative to the sowing date for yield or soil sulfate for protein. The early-reproductive period VPD affected all independent variables, namely RAU, yield, and seed protein. The elastic net algorithm successfully depicted some otherwise challenging empirical relationships to assess with bivariate associations in observational data. This approach provides inference about environmental variables while predicting N-fixation. The outcomes of this study will provide a foundation for improving the understanding of N-fixation within the context of sustainable intensification of soybean production.

## Introduction

Nitrogen (N) is the most significant nutrient demanded by soybean [*Glycine max* (L.) Merr.], supplied by both symbiotic fixation of atmospheric N_2_ and from soil minerals (Fabre and Planchon, 2000). Across the globe, most soybean systems do not rely upon N-fertilizer application (Herridge et al., 2008; Heffer et al., 2017), making N-fixation a critical process for enhancing the resilience of production. Soybean seed yield and protein concentration are related to the total plant N uptake (Fabre and Planchon, 2000; Ciampitti and Salvagiotti, 2018) and N-fixation accounts for 40 to 90% of N uptake (Ciampitti and Salvagiotti, 2018). The contribution of N-fixation to soybean nutrition is the result of an intricate relationship between host-bacteria symbiosis (Pauferro et al., 2010; Abou-Shanab et al., 2017) and the environment (Hungria and Vargas, 2000; Chalk et al., 2010; Santachiara et al., 2019). Unraveling N-fixation relationships with environmental conditions is crucial to achieving the goal of sustainable agriculture and economic competitiveness.

The relative abundance of ureides (RAU) and isotopic ^15^N-based methods can be used to quantitatively assess N-fixation (McClure et al., 1980; Unkovich and Pate, 2000). Although ^15^N-based methods provide a time-integrated N-fixation measure, they require complex implementation. For instance, the ^15^N natural abundance requires a non-leguminous or leguminous non-nodulating mutant reference plant (Unkovich et al., 2008), and errors arise from spatial variation of N pool and differences between crop and reference plant N acquisition patterns. The RAU method is straightforward with a less expensive determination (Unkovich et al., 2008) and is easy to upscale to a large number of field samples. Ureides (allantoin and allantoic acid) are the primary transport forms for fixed-N soybean (McClure and Israel, 1979), and the concentration in plant tissue correlates with the fraction of N derived from N-fixation (NDFN; Herridge and Peoples, 2002). The drawback of the ureide technique is that RAU may be affected by environmental and plant conditions without denoting *per se* N-fixation changes (Purcell et al., 2004). Nevertheless, RAU is widely used for measuring NDFN (Herridge and Peoples, 2002; Unkovich et al., 2008) and providing a reliable assessment across different environmental scenarios.

From a plant nutrition standpoint, uptake of mineral N is often described as having a negative relationship with N-fixation either in manipulative (Hungria et al., 2006; Salvagiotti et al., 2008; Tamagno et al., 2018) or observational studies (Schipanski et al., 2010). The N-fixation process is highly susceptible to drought conditions (Purcell et al., 2004; Kunert et al., 2016). High soil temperature (Lindemann and Ham, 1979), oxygen stress (Pasley et al., 2020), soil salinity, and other abiotic stressors also negatively affect N-fixation or nodulation (Chalk et al., 2010; Santachiara et al., 2019). Alternatively, adequate mineral availability of sulfur (Divito and Sadras, 2014; Miyatake et al., 2019), phosphorous (Collino et al., 2015), and iron (Rotaru and Sinclair, 2009) are likely to be beneficial for N-fixation in legumes. Although the abovementioned studies focused on the association of environmental variables with N-fixation, attempts to predict or model this biological process are scarce (Schipanski et al., 2010; Collino et al., 2015; Córdova et al., 2019).

Empirical models have encompassed only a few environmental factors and have experienced limitations when handling the multidimensionality of the N-fixation process. Schipanski (2010) reported that fixed-N is dependent on soil sand content, soybean genotype, and N associated with microbial biomass. In Argentina, soil phosphorous content, pH, and precipitation from sowing to flowering were related to N-fixation in high-yield environments, whereas mean air temperature during seed filling and precipitation during fallow were closely associated with N-fixation for low-yield (Collino et al., 2015). Both aforesaid examples used stepwise variable selection for low- and high-yield environments, but this procedure tends to inflate regression coefficients, increasing the likelihood of false-positive tests (Thompson, 2001). Moreover, plant biomass at physiological maturity alone is reported as a strong predictor (R^2^ > 0.83) of N-fixation (Córdova et al., 2019). However, using biomass to predict N-fixation along with environmental covariables brings the lack of independence between predictor variables.

Process-based models such as The Agricultural Production Systems Simulator (APSIM) and The Decision Support System for Agrotechnology Transfer (DSSAT) can simulate and predict fixation as a function of management, soil, weather, and cultivar, but the use of such models is complex and highly sensitive to input variables and constant parameters. In principle, these models simulate daily N-fixation as a function of plant growth rate and soil water/N/temperature limiting factors (e.g., APSIM; Pasley et al., 2020) or more mechanistically by simulating nodule mass and the cost of fixation (e.g., DSSAT; Boote et al., 2003). Besides specific estimates of N fixation using these models, simulations of this process at field scale are limited.

One of the challenges of accounting for multi-dimensional environment descriptors, particularly in observational data analysis, arises from collinearity [correlation between predictors, (Dormann et al., 2013)]. When variables are collinear, there is a greater likelihood of coefficient overestimation and poor identification of relevant predictors (McNeish, 2015). Regularization introduces a penalty on the coefficient estimates alleviating collinearity (Graham, 2003). The LASSO penalty (λ1, Least Absolute Squares, and Shrinkage Operator) implies the sum of absolute coefficient values and linearly shrinks coefficients to zero (Tibshirani, 1996). However, if two variables are highly correlated, LASSO arbitrarily selects one of the variables (Zou and Hastie, 2005). On the other hand, the RIDGE penalty (λ2) implies the sum of the squared coefficient values shrinking coefficients toward zero without reducing model dimensionality (Hoerl and Kennard, 1970). The elastic net procedure combines both penalties (LASSO and RIDGE) and provide a variable selection technique outperforming LASSO, mainly for large datasets (Zou and Hastie, 2005). Additionally, regularization supersedes stepwise selection because the former does not add or remove predictors repeatedly, while testing individual significances and increasing the chances of type I error (McNeish, 2015). Regularization introduces a penalty to every coefficient, and those with lower importance as predictors will be penalized to zero or close to zero providing a more parsimonious model (Graham, 2003). This method has also been previously employed to select and describe environmental variables associated with plant biophysical processes (Carter et al., 2018).

Therefore, we propose using the elastic net as a method for regularization and variable selection of a comprehensive set of environmental covariables with interpretable coefficients for improving our understanding of the N-fixation process in soybean. The central hypothesis is that soil, management, and weather covariables will enable satisfactory RAU predictions as an indicator of NDFN in different environments. Thus, we are interested in exploring the association between RAU, seed yield, and protein. Lastly, We also discussed the biological importance of the most relevant environmental covariables with RAU within the context of the current literature.

## Materials and Methods

### Experimental Design and Treatments

During the 2018 and 2019 growing seasons, 21 field experiments were conducted at 11 research stations in six of the US states (Arkansas, Iowa, Indiana, Kansas, Minnesota, and South Dakota). Nitrogen management strategies were the main treatment factor (e.g., N fertilization and reinoculation), although without treatment balance across experiments. Sowing date and genotype were also tested in some of the studies. Variation in soil properties in the entire dataset is attributed to the spatial distribution of the experiments across site-year combinations. Weather parameters varied due to the site, year, sowing date, and season length, thus generating 46 combinations of different environmental conditions during crop growth, hereafter called “environment.”

The database comprises field experiments with different treatment structures. Nitrogen management strategies (the manipulative treatment in all field experiments) were classified as control, N fertilization, and reinoculation. The N-fertilization group included all rates (from 10 to 170 kg ha^−1^), sources [ammonium sulfate (AMS, 21–0–0-24, N-P-K-S), and urea ammonium nitrate (UAN, 28–0–0-0, N-P-K-S)], and timings of N fertilization [from pre-sowing to the seed filling period (R5; Fehr et al., 1971)]. The reinoculation group comprised treatments with the application of surface banded *Bradyrhizobium japonicum* (Terramax Inc., Bloomington MN, USA) with a dose of 1.3 L ha^−1^ diluted in water (volume of 200–280 L ha^−1^) during the vegetative and the reproductive stage. Seed inoculation was applied in both the N-fertilization and reinoculation groups. Supplementary Table S1 contains a complete description of the treatments. The control group included treatments presumed to not affect N-fixation (e.g., pesticide application) in addition to the designated control. A randomized complete block design (RCBD) was utilized with three to five replications. All replications were averaged by unique location, year, sowing date, maturity group (MG), and management category and generated 95 environments by treatment combinations. Field studies were sown from May 7th to June 27th of each growing season, and genotypes included MG ranging from 1.0 to 4.6 (Mourtzinis and Conley, 2017; Supplementary Figure S1).

### RAU, Seed Yield, and Protein

Main stems were collected during early seed filling (R5 stage) from 10 consecutive soybean plants to compose a plot sample. The samples were dried at 65°C in an air-forced oven until constant weight and then ground in a micro mill (60-mesh screen). Xylem N solutes were extracted from 0.3 g of dry stem tissue with ethanol (99.9%) and 0.1 M phosphate buffer (Hungria and Araujo, 1994). Dry tissue extracts were measured by colorimetric determination of ureides (Vogels and van der Drift, 1970) and nitrate analysis *via* the salicylic acid method (Cataldo et al., 1975). Ureides and nitrate concentration (μmol g^−1^) were used to calculate the RAU-N (%) as a proxy of the fraction of N derived from symbiotic N-fixation following the method described by Unkovich et al. (2008):

(1) $$\mathrm{RAU}=\left[4\times \left[\mathrm{ureide}\right]\right]/\left[4\times \left[\mathrm{ureide}\right]+\left[{\mathrm{NO}}_{3}{}^{-1}\right]\right]$$

Other N solutes (e.g., amino acids) were not considered for RAU calculation. For more details on the ureide analysis, review Moro Rosso et al. (2021).

At maturity (R8), the center rows of each plot were mechanically harvested to estimate yield. A seed sample was collected for moisture and protein concentration determination. Seed yield was adjusted to 130 g kg^−1^ of water content. Seed protein analysis was accomplished by grinding the material to a 0.1 mm final particle size and estimating dry basis protein concentration (g kg^−1^) with near-infrared spectroscopy (NIR) using the Perten DA7200 Feed Analyzer (Perten Instruments, Stockholm, Sweden; Fontaine et al., 2001; Rotundo et al., 2016). The reflectance of the seed tissue was measured between 1,000 and 2,500 nm wavelength and normalized to the ceramic reference plate holding the material. The method of calibration is based on the regression from Honigs (1985) and retrieved cross-validation R^2^ of 0.9 for seed protein concentration.

### Environment Covariables and Crop Modeling

Environment covariables were composed of soil and weather factors. A selected number of soil attributes were directly measured at each location, including S-sulfate-S (SO_4_), nitrate-N (NO_3_), and cation exchange capacity (CEC) from the 0–0.15 m soil depth. Additional soil variables [soil organic matter (SOM), clay, silt, and sand content] were acquired from the POLARIS database (Chaney et al., 2016). Similarly, daily weather data were retrieved from the GridMet database (Abatzoglou, 2013) from sowing to harvest time at each location. Two additional indices were calculated: the Shannon diversity index (SDI) of precipitation (Bronikowski and Webb, 1996) and organic matter resilience to mineralization (OMM; Pieri, 1995). A complete overview of soil and weather variables is presented in Table 1.

**Table 1:** Crop, soil, and weather variable description, units, means, and range of observations.

Variable	Description	Unit	Mean, [min, max]	Group
MG	Maturity group	-	3.0, [1.0, 4.6]	Crop
S.Phe	Ureide sampling relative stage^1^	-	1.6, [1.3, 1.9]	Crop
S.Length	Season length	days	98, [75, 112]	Crop
Sowing	Sowing date of the treatments	day of year	151, [126, 177]	Crop
CEC	Soil cation exchange capacity	cmol_c_ dm^−3^	17, [3, 27]	Soil
Clay	Clay relative content	%	20, [7, 31]	Soil
N-NO_3_	Soil nitrate content before crop sowing	mg dm^−3^	6.0, [1.0, 10.6]	Soil
OMM	Organic matter mineralization	%	0.04, [0.01, 0.07]	Soil
pH	Soil pH	-	6.5, [5.6, 7.1]	Soil
Sand	Sand relative content	%	29, [9, 50]	Soil
Silt	Silt relative content	%	47, [34, 63]	Soil
S-SO_4_	Soil sulfate content before crop sowing	mg dm^−3^	7.7, [0.9, 21.3]	Soil
SOM	Soil organic matter	g kg^−1^	27, [6.4, 48]	Soil
ET_0_	Cumulative reference evapotranspiration	mm	440, [337, 602]	Weather^3^
Hum	Daily mean relative air humidity	kPa	2.1, [1.7, 2.4]	Weather
Prec	Cumulative rainfall precipitation	mm	438, [170, 805]	Weather
Rad	Cumulative solar radiation	MJ m^−2^	2,186, [1,680, 2,595]	Weather
SDI	Precipitation evenness: SDI^2^	-	0.66, [0.53, 0.72]	Weather
D.str	Drought stress: Cumulative ET reduction	mm	4.5, [0.0, 19.4]	Weather
T.Amp	Daily mean temperature amplitude	°C	11, [10, 12]	Weather
Tmean	Daily mean temperature	°C	22.9, [19.9, 25.6]	Weather
VPD	Daily mean vapor pressure deficit	kPa	0.78, [0.64, 1.08]	Weather

1 Relative phenological stages according to SoySim. Values range from 0 to 2, where 1 and 2 represent R1 and physiological maturity, respectively.

2 SDI: Shannon diversity index, where 1 denotes an uneven distribution and 0 implies a skewed distribution.

3 Weather was segmented in vegetative (Wv), pre-seed filling (Wr), and seed-filling (Ws).

Crop covariables included management features presenting relevant variation across environments as genotype MG, season length, sowing date, and stem sampling date for RAU assessment. The SoySim crop model (Setiyono et al., 2010) was used to estimate season length from VE (emergence) to R7 (physiological maturity) since locations reported only planting and harvesting dates. Also, the relative phenological scale from SoySim was obtained to select sampling dates performed during R5 to R6 growth stages. Stem samples outside this range were removed from the database. Nevertheless, we accounted for the relative phenological stage of RAU sampling as a crop covariable in the model. Other covariables related to management (e.g., row spacing, plant density, and rotation scheme) were not considered because of the lack of variation across observations.

Drought stress was estimated using the Soil, Water, Atmosphere, and Plant model (SWAP; Kroes et al., 2008). The water retention curve parameters (ksat, θ_sat_, θ_res_, m, and α) were extracted from a level of 0.05 m to a 0.6 m soil depth (Chaney et al., 2016). Crop initialization was set up according to the SoySim estimated VE and R7 dates. Crop evapotranspiration (ET_c_) was given by the relationship between crop coefficient and the Penman-Monteith reference evapotranspiration (ET_0_; Allen, 1998). Drought stress was defined as a reduction in crop transpiration when soil water potential decreased below −80 kPa at low ET_c_ or −50 kPa at high ET_c_ conditions (Clemente et al., 1994; Feddes et al., 2001). Transpiration reduction ranged from 0 (no drought) to 100% (severe drought stress).

Finally, simulated phenology was used to summarize all weather variables for different periods of the crop development: (1) vegetative [v, from VE to pre-flowering (R1)]; (2) reproductive pre-seed filling [r, from R1 to pod setting (R4)]; and (3) reproductive seed filling (s, from R5 to R7). Table 1 presents all crop and environment descriptors, with respective units, range, and statistics used to summarize weather variables (e.g., mean, sum).

### Statistical Model and Regularization

Data analysis was designed to explore the relationship between environment and plant covariables for each response variable (RAU, yield, and protein) while making out-of-bag predictions using a simple linear model. The Pearson’s correlation coefficient (r) was used first to filter out significant (*p* < 0.05) highly correlated (|r| > 0.75) covariables with similar biological meaning. Sand, silt, and clay were highly correlated, and due to the low correlation between sand and soil organic matter, clay and silt were dropped from the analysis. Similarly, soil organic matter and organic matter mineralization were correlated and only the former was included. Relative air humidity was removed due to correlation with mean temperature, and reference evapotranspiration was excluded due to a VPD correlation (Supplementary Figure S2). We have selected 35 (33 continuous and the binary N-fertilization and reinoculation) from a total of 40 initially observed covariables.

Subsequently, a single slope was proposed for each covariable. The models presented a random intercept for the site group (each site), in addition to the fixed effects. Other random groups (e.g., year) were not considered to maintain model simplicity (Matuschek et al., 2017). The models were fitted in the *R software* (R Core Team, 2019) using the *MMS* package (Rohart et al., 2014). The *lassop* function was used to fit the elastic net regression models based on expectation-maximization algorithms (Rohart et al., 2014). The arguments for the *lassop* function are λ and α; λ controls the overall amount of penalty, and α controls the proportion of λ1 and λ2. Both can be determined *via* cross-validation. Based on preliminary analysis and computational efficiency, the mixing parameter (α) and penalty parameter (λ) for all full models were set at 0.50 (Friedman et al., 2010). The *lmme* function was used to obtain the maximum likelihood of the coefficients without the penalty (no penalty model) and fit an intercept-only model (null model, the intercept is not shrunk).

### Nested Cross-Validation and Reduced Model

Developing predictions for RAU, yield, and protein, a nested cross-validation scheme was proposed to estimate (1) the hyperparameter λ (inner loop) and (2) overall predictability (outer loop). Considering a relatively small database (Zhang and Ling, 2018), a 10-fold cross-validation was implemented in the inner loop, while a 20-fold cross-validation was designed for the outer loop (Correndo et al., 2021). The median root mean squared error (RMSE) was used to select the λ hyperparameter from a linear sequence of 90 log-transformed values. The prediction performances were evaluated by RMSE, mean absolute error (MAE), relative RMSE (RRMSE), and R^2^ using the outer loop. The full model penalized coefficients were grouped using the K-means algorithms based on their absolute magnitude to identify covariables with a stronger association with the predicted variables. Finally, a reduced model was proposed only for RAU using the covariables with the strongest associations found by the full model. The same methods implemented for the full model were repeated for the reduced model (cross-validation). The partial dependency plots were presented to explore the relationship between RAU and the most important covariables.

## Results

### Data Description

**Figure 1:**
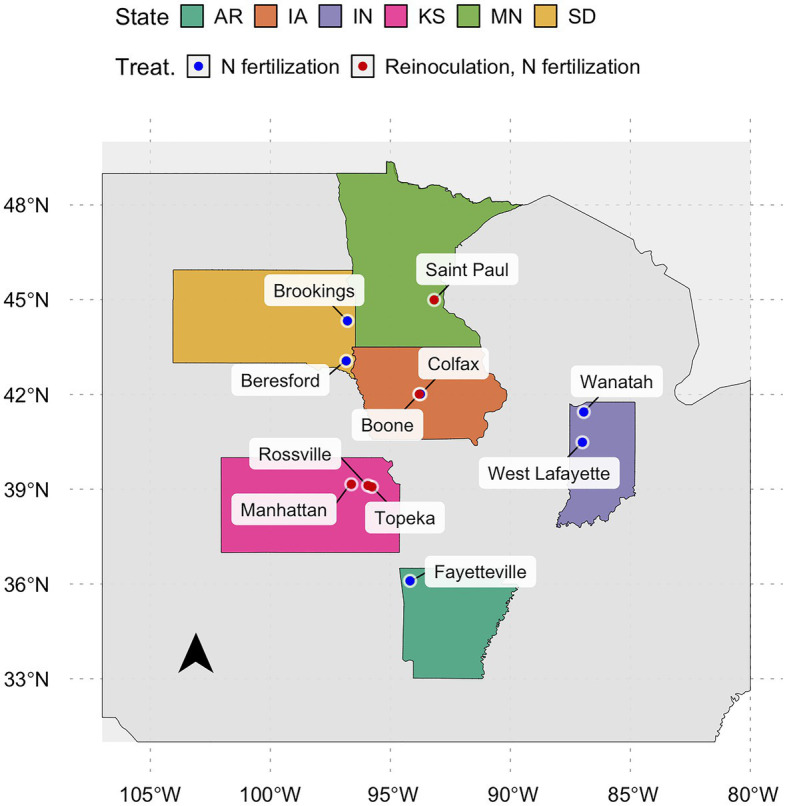
Locations of the field studies during the 2018 and 2019 growing seasons. Red points are environments with control, reinoculation, and N-fertilizer; and blue points are environments with only control and N-fertilizer.

In total, 95 observations from environments containing N-fertilizer, reinoculation, or both treatments composed the database (Figure 1). In temporal terms, 34 and 66% of the observations came from the 2018 and 2019 growing seasons, respectively. Observed RAU values ranged from 60% (Saint Paul, MN) to 98% (Boone, IA and Fayetteville, AR; Supplementary Figure S1). Seed yield ranged from 2.9 to 5.6 Mg ha^−1^ with a mean of 4.0 Mg ha^−1^. Yield observations were evenly distributed across states, with Fayetteville (AR) displaying the highest productivity. Seed protein concentration had a narrow range from 376 g kg^−1^ (Boone, IA) to 429 g kg^−1^ (Rossville, KS), with an overall mean of 398 g kg^−1^.

The observed range and density estimation of covariables selected to take part in the full model are described in Supplementary Figure S1. There was a relatively wide range of soil S-sulfate (from 0.9 to 21.3 mg kg^−1^), CEC (from 3 to 27 cmol_c_ dm^−3^), and organic matter (from 6 to 48 g kg^−1^) observed across field studies. During the vegetative period, meteorological parameters presented a more narrow range across environments than pre-seed filling and seed filling. Cumulative radiation during pre-seed filling averaged 849 MJ m^−2^, whereas seed filling averaged 599 MJ m^−2^. Precipitation presented similar ranges between vegetative (from 40 to 283 mm), pre-seed filling (from 34 to 354 mm), and seed filling (from 37 to 337 mm) stages. No drought stress was estimated during vegetative growth, and only mild drought was found during pre-seed filling (x̄ = 1.1%); therefore, potential drought effects in early- and mid-seasons were removed from the full model. Moreover, strong drought stresses were observed during seed filling with an average of 12.5 mm across environments and ranged from 0% (many locations) to 44% (West Lafayette, IN) of transpiration reduction.

### Full Model Tuning and Precision

**Figure 2:**
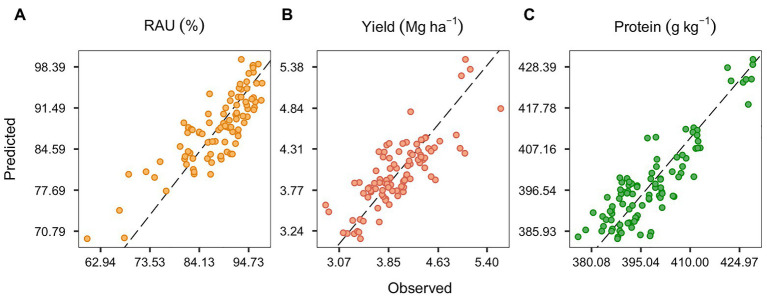
Predicted and observed data for the relative abundance of ureides (RAU; **A**), seed yield **(B)**, and seed protein concentration **(C)**. Elastic net regression model considering all the environment, crop, and management covariables (full model).

The observed and predicted values of RAU, yield, and protein using the full set of covariables are depicted in Figure 2. The selected λ_1_ penalties for the RAU, yield, and protein full models were 0.0067, 0.0925, and 0.0014, respectively, based on the least RMSE median of the nested cross-validation in the out-of-bag procedure (Supplementary Figure S3). It is worth noting that introducing bias through the elastic regularization methods (weighing RIDGE and LASSO penalties) improved predictive accuracy in models for both yield and RAU. However, the protein model performed similarly to the no-penalty model (Supplementary Figure S3), suggesting a possible underfitting for this variable. The full model RMSE was 3.9% for RAU, 0.3 Mg ha^−1^ for yield, and 5.4 g kg^−1^ for protein concentration. Other metrics for model precision (MAE and RRMSE) are shown in Table 2. For all predicted variables, the inclusion of soil, crop, and weather covariables in the full model improved the precision over the null model (intercept only).

**Table 2:** Full models precision and accuracy metrics for the RAU, yield, and protein concentration.

**a) Model metrics**					
	Model	MAE	RMSE	RRMSE	R^2^
RAU (%)	Null	6.1 (±2.8)^1^	7.0 (±3.3)	8.1 (±4.6)	
	Full	3.4 (±1.5)	3.9 (±1.6)	4.5 (±2.2)	0.66 (±0.3)
Yield (Mg ha^−1^)	Null	0.4 (±0.1)	0.5 (±0.2)	11.9 (±4.2)	
	Full	0.2 (±0.1)	0.3 (±0.1)	6.9 (±3.2)	0.68 (±0.3)
Protein (g kg^−1^)	Null	10 (±3.3)	12 (±3.8)	2.9 (±0.9)	
	Full	4.8 (±1.6)	5.45 (±1.8)	1.4 (±0.4)	0.70 (±0.3)
**b) Random effects variance**					
		Environment	Residual		
RAU	Null	31.5 (±3.1)	26.6 (±1.4)		
	Full	0 (±0)	9.2 (±0.5)		
Yield	Null	0.10 (±0.01)	0.13 (±0.01)		
	Full	0.001 (±0)	0.01 (±0.01)		
Protein	Null	118.5 (±4.6)	56.5 (±1.7)		
	Full	0 (−±0)	16.1 (±0.7)		

Mean absolute error (MAE), root mean squared error (RMSE), relative root mean squared error (RRMSE), and coefficient of determination (R^2^). (a), Full model variance partition; (b) Random effects variance.

1 Median value followed by the standard deviation from the cross-validation procedure.

The variance partitioned to the site random effect accounted for a substantial partition of the null model variance. The variance was reduced to zero when the full set of weather, soil, and crop covariables were added to the training dataset (Table 2). The variance from the environment was 31.5%, 0.10 Mg ha^−1^, and 118.5 g kg^−1^ for RAU, yield, and protein, respectively. The full model residual variances were also reduced (9.2%, 0.01 Mg ha^−1^, and 16.1 g kg^−1^ for RAU, yield, and protein, respectively) compared with the null model (26.6%, 0.13 Mg ha^−1^, 56.5 g kg^−1^ for RAU, yield, and protein, respectively).

### Covariables Importance

**Figure 3:**
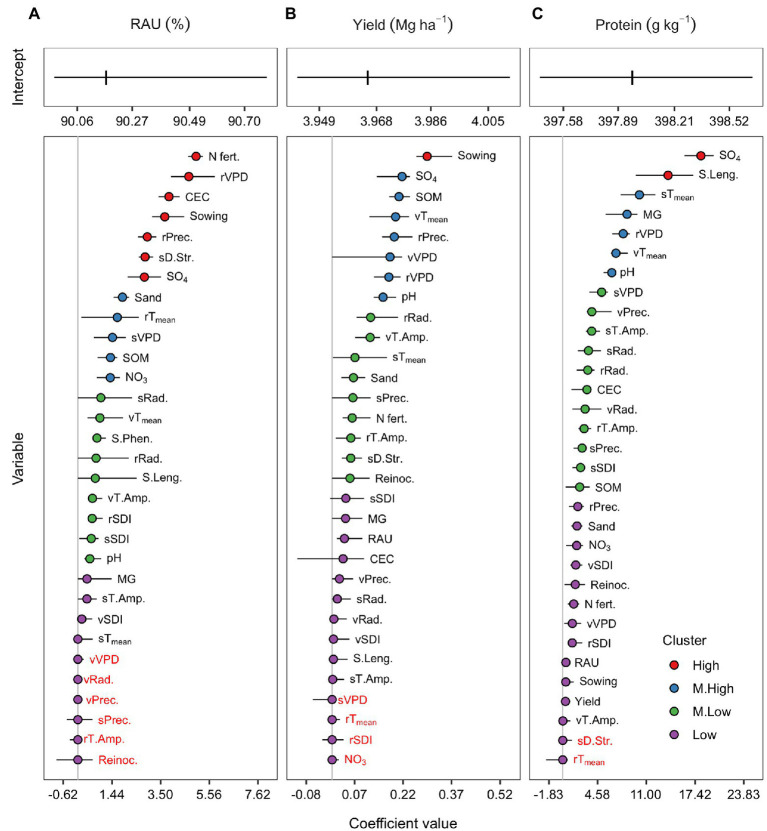
Centered and scaled covariables coefficients of the full model for the RAU **(A)**, seed yield **(B)**, and protein concentration **(C)**. Coefficients were grouped in high (red), medium-high (blue), medium-low (green), and low (purple) magnitude clusters by the k-means algorithm. Coefficients with red letters were shrunk to zero by the elastic net regularization process. Points represent the median from the 20 training-test combinations, while horizontal lines range from the minimum to the maximum coefficient values across sets.

The coefficients for the full model for RAU, yield, and protein were sorted according to their magnitude, regardless of the direction and grouped by the K means clustering algorithm (Figure 3). Nitrogen fertilization, VPD, precipitation during pre-seed filling, soil CEC, sowing date, drought stress in the seed filling, and soil sulfate were the features presenting a stronger RAU association (Figure 3A). The medium-high magnitude group contained soil sand, pre-seed filling temperature, seed filling VPD, SOM, and soil nitrate content. The medium-low and low clusters gathered features with less importance but were still associated with RAU. These were soil pH, season length, seed filling radiation, and SDI, among others. Notably, the coefficient of Phe was not shrunk to zero in most of the training-test sets, despite the relatively narrow sampling interval. During vegetative crop development, weather covariables showed overall low importance for RAU predictions. They were primarily grouped in the low cluster or shrunk to zero (except mean temperature and thermal amplitude). Interestingly, reinoculation treatment was not associated with the RAU, as suggested by its cancelation coefficient by the elastic net.

The main reason for building predictive models for yield and protein was to evaluate the importance of RAU to these agronomic traits. Overall, RAU presented a median coefficient different from zero for both yield and protein models; however, it was clustered in the low magnitude group (Figures 3B,C). Other plant and weather features presented greater importance than RAU for predicting yield and protein. The covariable with the highest importance in the yield model was the sowing date (Figure 3B). In contrast, soil sulfate and season length were the few features with a high magnitude cluster for the protein model (Figure 3C).

The introduction of penalties throughout the elastic net procedure changed the coefficient magnitude but did not alter relative importance and direction of each predictor. Overall, the non-penalized models (equivalent to the maximum likelihood estimate) presented coefficients with greater magnitude than the penalized models (Supplementary Figure S4). The coefficient direction (positive or negative) in the non-penalized model was usually the same after penalty. Among the penalized models, the increase of *α* (0 to 1) toward LASSO subsided the magnitude of the coefficients.

### Reduced Model

We used the regularization method in the full model with the adjunct goal of an automatic variable selection and of reducing the dimensionality of the RAU predictive model. Although the elastic net canceled 6 out of 31 covariables (Figure 3), the model was not sparse enough for a parsimonious agronomic interpretation. Thus, we proposed a reduced model with only features from the high and medium-high importance clusters, comparing the new model performance to the full model.

**Figure 4:**
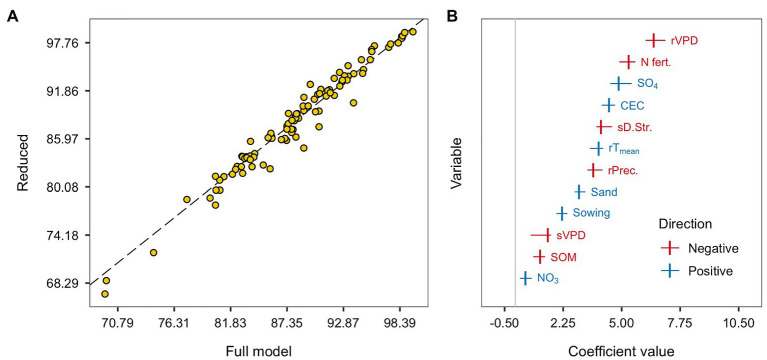
The overlaying of predicted values of the full and reduced model for the RAU. The dashed black line indicates the 1:1 relation **(A)**. Centered and scaled covariables coefficients of the reduced linear regression model. Colors indicate the coefficients directions: negative are reds and positive are blues. Points represent the median from the 20 training-test combinations, while horizontal lines range from the minimum to the maximum coefficient values across sets **(B)**.

The overlaying of observed and predicted values of the reduced and the full models for RAU prediction are presented in Figure 4A. The reduced non-penalized model showed similar predictive performance. The residual variance increased by 6.4% in the reduced model, but the RMSE and R^2^ remained in the same range, with a median of 3.4 (±1.4) and 0.73 (±0.26), respectively, in the reduced model and a median of 3.9 (±1.6) and 0.66 (±0.3) in the full model. As expected, the imposition of shrinkage to the reduced model little affected the coefficient magnitudes, justifying the choice of the non-penalized regression. The reduced and full models present a slightly different order among the importance for each covariable. The VPD during pre-seed filling became the most important feature, followed by N-fertilization, soil sulfate, CEC, drought stress during the seed filling, pre-seed filling temperature and precipitation, sand, and sowing date (Figure 4B). Covariable coefficients swapping positions in the magnitude scale was expected when a model with fewer predictors refits the original dataset.

**Figure 5:**
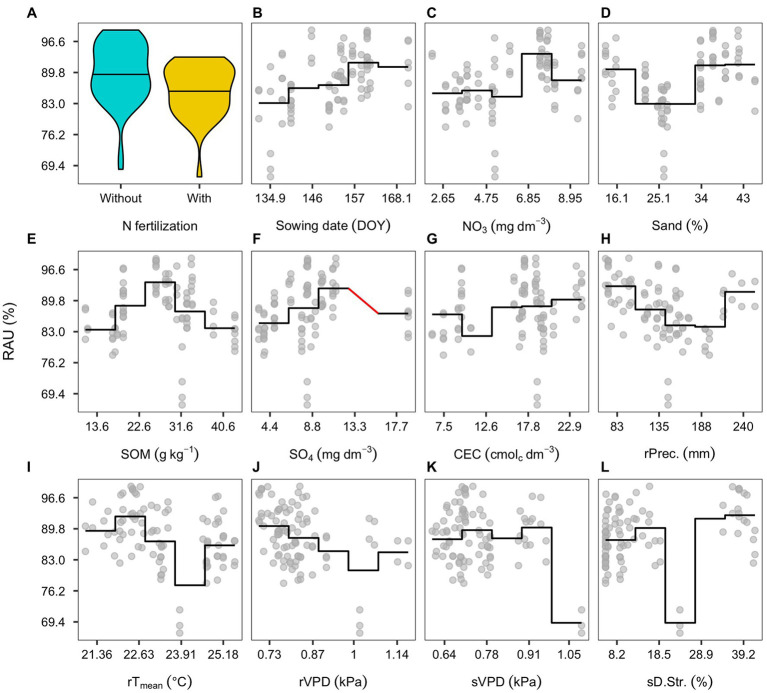
Partial dependency plots of environmental effects affecting RAU: Nitrogen (N)-fertilization **(A)**; sowing date (DOY; **B**); soil nitrate concentration **(C)**; sand concentration **(D)**; soil organic matter (SOM; **E**); soil sulfate concentration **(F)**; soil cation exchange capacity (CEC; **G**); precipitation during pre-seed filling **(H)**; radiation during pre-seed filling **(I)**; vapor pressure during pre-seed filling **(J)**; vapor pressure during seed filling **(K)**; drought stress during seed filling **(L)**. Solid lines represent a segmented mean for seven intervals comprised within the variables range. Red lines represent the absence of observations.

Partial dependency plots describe the association between predicted RAU, plant, and environmental features in the reduced model. The presence of N-fertilization showed 5% average reduction in the predicted RAU (Figure 5A). The sowing date was positively linked sto RAU until around the second week of June (158 DOY; Figure 5B). The pre-season soil nitrate status presented a wide variation but an overall positive association (Figure 5C). The soil sand content and RAU were positively associated mainly with sand content levels above 34% (Figure 5D). Similarly, to sand, the association of SOM with RAU differed across environments. Below sand content of 22.5 g kg^−1^, the association was not clear, but above 30 g kg^−1^, there was a negative trend (Figure 5E). Sulfate concentration in the soil presented a positive coefficient with RAU, mostly visualized from 1 to 11 mg dm^−3^ (Figure 5F). Soil CEC was also positively associated with RAU, although showing a substantial variation between 16 and 18 cmol_c_ dm^−3^ (Figure 5G). Cumulative precipitation during pre-seed filling was negatively associated with RAU, with erratic predictions with a large amount of precipitation (above 200 mm; Figure 5H). The daily mean temperature during pre-seed filling presented a positive association with RAU as depicted in the reduced model. However, this relationship was not clearly visible in the dependency plot (Figure 5I). During pre-seed filling and seed filling, VPD showed a negative relationship with RAU; in the pre-seed filling, it was mostly from the lower limit (0.7) to around 1.0 (Figure 5J), and in seed filling, it depicted a lower magnitude (Figure 5K). Finally, drought stress during seed filling presented a negative coefficient despite the erratic behavior throughout the range of levels (Figure 5L).

## Discussion

This study included weather, soil, and management covariables from 95 environmental conditions uncovering associations and predicting RAU. Our results expand previous findings on qualitative associations (Chalk, 2000; Hungria and Vargas, 2000) and effect sizes of independent environmental factors on RAU (Santachiara et al., 2019). Using multivariate analysis, this approach goes beyond previous reports since it considers many variables while dealing with their natural collinearity commonly related to biological processes (Dormann et al., 2013). It is noteworthy that some directions captured by the reduced model are not easily visualized in the partial dependency plots (Figure 5). Observational datasets are relatively easy to collect, but they carry limitations that the statistical analysis cannot overcome. Notably, we do not account for all possible factors related to RAU, yield, or seed protein concentration, whereas a lurking variable could improve the model and change some of the associations found by our analysis. Also, causation relationships should not be assumed. Although we followed a nested cross-validation scheme, an independent dataset did not validate our model predictions. Diligence must be considered for further extrapolations, and controlled studies must explore the environmental associations, which are not previously described.

Both RAU and yield were connected to some common covariables (i.e., VPD, soil sulfate content, SOM, and pre-seed filling precipitation), suggesting the interplay of a growth-intermediated process and N-fixation (Chalk, 2000). N-fixation is regulated by the strength of the plant N-sink (Schulze, 2004) and correlated to aboveground biomass (Tamagno et al., 2018; Córdova et al., 2019). Similarly, N-fixation and protein concentration are slightly correlated (Fabre and Planchon, 2000) and are prone to interactions with the environment (Assefa et al., 2019). It is worth noting that RAU had a narrow range of values concentrated near the upper boundary of previous reports (Carciochi et al., 2019; Moro Rosso et al., 2021). The fact that RAU was grouped with low importance for predicting yield (and protein) indicates that improvement in N-fixation in such environments would lead to a marginal increase to these traits. A similar outcome was obtained by Archontoulis et al. (2020) with N-fixation having low sensitivity to predict soybean yield.

Nitrogen fertilization was associated with RAU reduction. However, the diversity of environments and the N-fertilizer strategies provided a smaller decline (~5%) than some previous studies with standard treatments (Purcell et al., 2004; Tamagno et al., 2018; Moro Rosso et al., 2021). Soil organic matter was also negatively associated with RAU, particularly in environments with relatively high SOM content scenarios (Figure 5E), because of the continued N mineralization supply throughout the season. The positive relationship between RAU and sand content (Figure 4B) confirms the results of Schipanski et al. (2010) and Tamagno et al. (2018) due to the likely indirect effect of oxygen depletion under water excess on N-fixation (Schipanski et al., 2010). Finally, the positive association between soil sulfate and RAU (Figure 4B) is an indication that S directly enhanced N-fixation in legumes (Divito and Sadras, 2014) or indirectly had an effect on plant growth, promoting yield, and seed protein concentration (Figure 3A). The CEC, a variable that is a proxy for soil N fertility, was strongly associated with RAU (Figures 3A, 4B) but not with seed yield (Figure 3B), suggesting that CEC is a relevant soil variable related to N-fixation directly without the mediation of plant growth. To the extent of our knowledge, such an association has not been reported. However, others N-cycle organisms, such as the free-living ammonia-oxidizing bacteria, were also associated with soil chemical fertility (Ciccolini et al., 2016). Therefore, regularization is also a tool for suggesting hypothesis testing (Sheetal et al., 2020), which future controlled studies should achieve.

On the other hand, some of our model coefficients exposed unusual relationships, for example, a trade-off between RAU and sowing date, even though early sowing time was linked to high yields (Supplementary Figure S4). This association is not supported by the positive correlation between N-fixation and aboveground biomass (Córdova et al., 2019). In addition, we found a pre-season soil nitrate positively related to RAU potentially improving early growth and development. Although these signals are significant within our observations, they might be the product of a lurking covariable promoting high N availability late in the season. The downregulation of RAU in early sowing may be explained by a supplemental N source from SOM mineralization during the late season (Blesh, 2019), negatively related to the pre-season nitrate content. Moreover, yield and protein concentration were both associated with N-fertilization in a higher cluster than RAU (Figures 3B,C), suggesting a plausible crop response to supplemental N (Mourtzinis et al., 2018; Tamagno et al., 2018). Our results indicate that seasonal SOM mineralization and, therefore, variations on indigenous N supply could become a strong predictor of soybean N-fixation.

As drought stress increased during seed filling, the RAU decreased, contrasting previous reports connecting drought with greater ureide concentration (Purcell et al., 1998, 2004). Drought stress is detrimental to N-fixation, and RAU concentration increases in the petiole and leaflets due to the reduction of ureide catabolism (Purcell et al., 1998). However, our results are based on RAU-derived from the main stem (without petioles), suggesting that RAU from different plant sections may respond differently to this stress condition. Conversely, the negative association between VPD during pre-seed filling and RAU is unlikely to be related to water deficit. The VPD levels were below the threshold, leading to a reduced transpiration rate (~2.5–3.0 kPa; Devi et al., 2015; Grossiord et al., 2020). The fact that one of the most important predictors (VPD during pre-seed filling) presents such high seasonal variability indicates that multiple RAU measurements could improve our understanding of factors affecting N-fixation. Nevertheless, future research should also explore how environmental covariables interact with the seasonal N fixation process, and predictive models could consider more than one-time point sampling or time-integrated N-fixation indicators.

## Conclusion

Different weather, soil, and plant covariables, including N-fertilization, pre-sowing soil sulfate concentration, soil CEC, drought stress, precipitation, sand content, and VPD during the reproductive stages, were necessary to predict RAU in soybean systems using an empirical model.

The elastic net regularization process coped with covariable collinearity while providing interpretable coefficients. Our model depicted some expected associations between RAU and environmental conditions while finding new relationships for future investigations. The associations between RAU and yield or protein were important but with a magnitude than other factors.

Future research may focus on the seasonal variation of N-fixation and its relationship with environmental conditions.

## Data Availability Statement

The raw data supporting the conclusions of this article will be made available by the authors, without undue reservation.

## Author Contributions

AB: data curation, analysis, visualization, original draft, and review and editing. LM: methodology, data curation, analysis, visualization, original draft, and review and editing. LP: data curation, methodology, and review and editing. SN, SC, PK, SA, AB, and DD: data curation, methodology, and review and editing. IC: conceptualization, methodology, original draft, review and editing, funding, and project administration. All authors contributed to the article and approved the submitted version.

### Conflict of Interest

The authors declare that the research was conducted in the absence of any commercial or financial relationships that could be construed as a potential conflict of interest.
