# Supplementary material for: Environmental Factors Associated With Nitrogen Fixation Prediction in Soybean
Source: Front Plant Sci. 2021 Jun 15;12:675410. doi: 10.3389/fpls.2021.675410 (PMC8239404; doi:10.3389/fpls.2021.675410)
Supplement: Supplementary file 1 [file Data_Sheet_1.PDF]

Supplementary Material

Supplementary Tables

Supplementary Table 1. N-fertilizer and inoculation treatments description.

Location	Nitrogen treatments (kg ha^{-1})	Inoculant treatments
Brookings, SD	55 at sowing	not present
Beresford, SD	10 at sowing and 45 at V4	
	10 at sowing and 45 at R3	
	10 at sowing, 45 at V4, and 45 at R3	
Fayetteville, AR	90 at R2	not present
	90 at R5	
Wanatah, IN	10 at pre-sowing	not present
West Lafayette, IN	10 at pre-sowing	
	45 at pre-sowing	
	45 at R3	
	20 at pre-sowing	
	45 at V4	
	45 at V4 and 45 atR3	
Saint Paul, MN	45 at R2	Seed inoculation and reinoculation at R1
	45 at R2 and 45 at R5	Seed inoculation and reinoculation at R1 and R3
	45 at R5	Seed inoculation and reinoculation at R3
	30 at R5, 30 at R5.5, and 30 at R6	
Boone, IA	18 at sowing	Seed inoculation and reinoculation at R1
	170 at sowing	
Colfax, IA	225 at R3	not present
Manhattan, KS	20 at sowing	Seed inoculation and reinoculation at R2
Rossville, KS	110 at sowing, 110 at R1, and 110 at R5	Seed inoculation and reinoculation at V4
		Seed inoculation and reinoculation at R1
Topeka, KS	20 at sowing	Seed inoculation and reinoculation at R1

Supplementary Figures

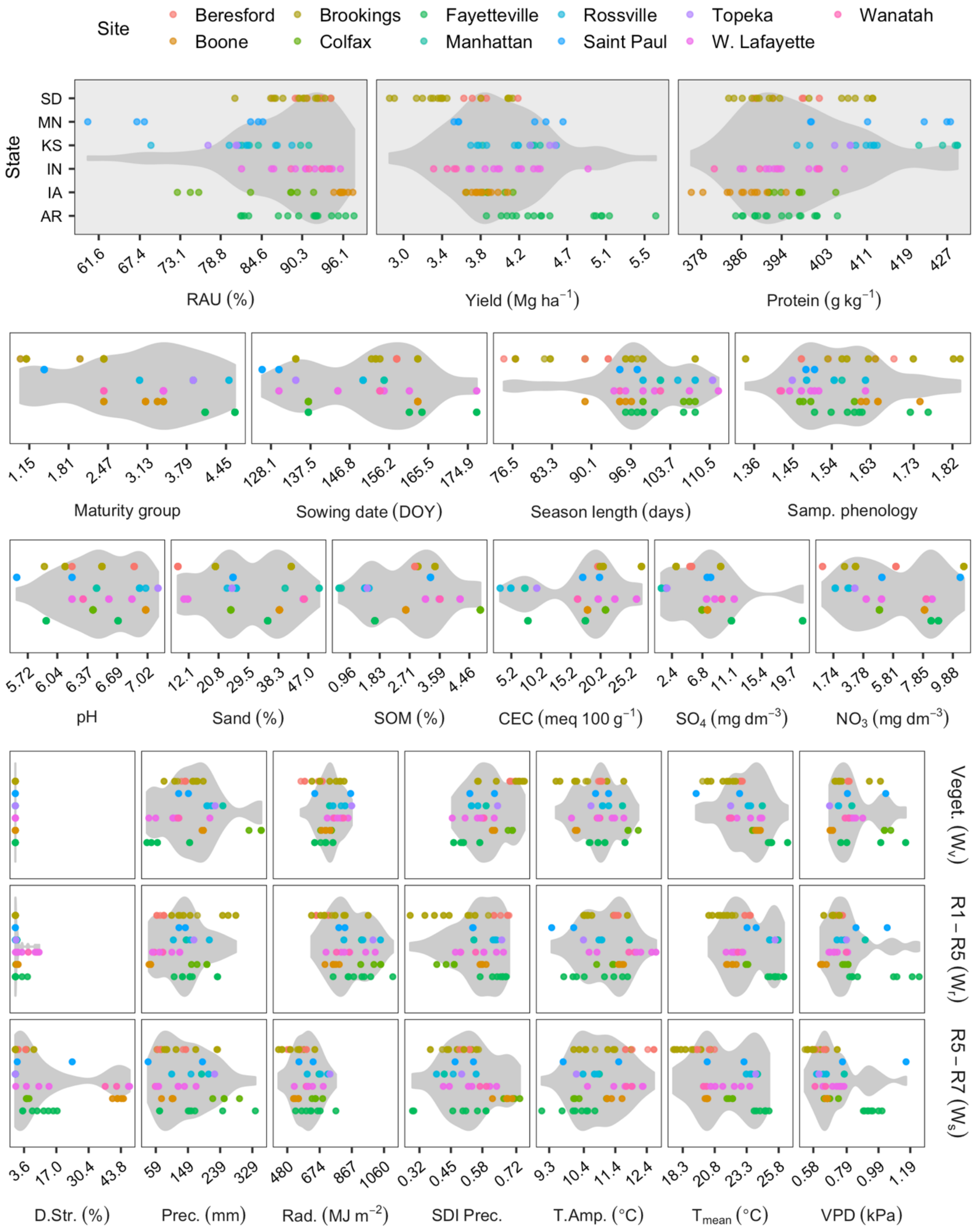

Supplementary Figure 1. Representation of observed variables (RAU, yield, and seed protein concentration) and environmental covariables. Point colors represent the field experiments per location. Violin plots show density estimation and population probability across all sites. Weather

covariates are segmented in vegetative (W_v), pre-seed filling (W_r) and seed filling (W_s) periods. Hum (relative air humidity); Prec (rainfall precipitation); Rad (solar radiation); SDI (precipitation evenness); D.str (drought stress); T.Amp (air temperature amplitude); Tmean (air temperature); VPD (vapor pressure deficit)

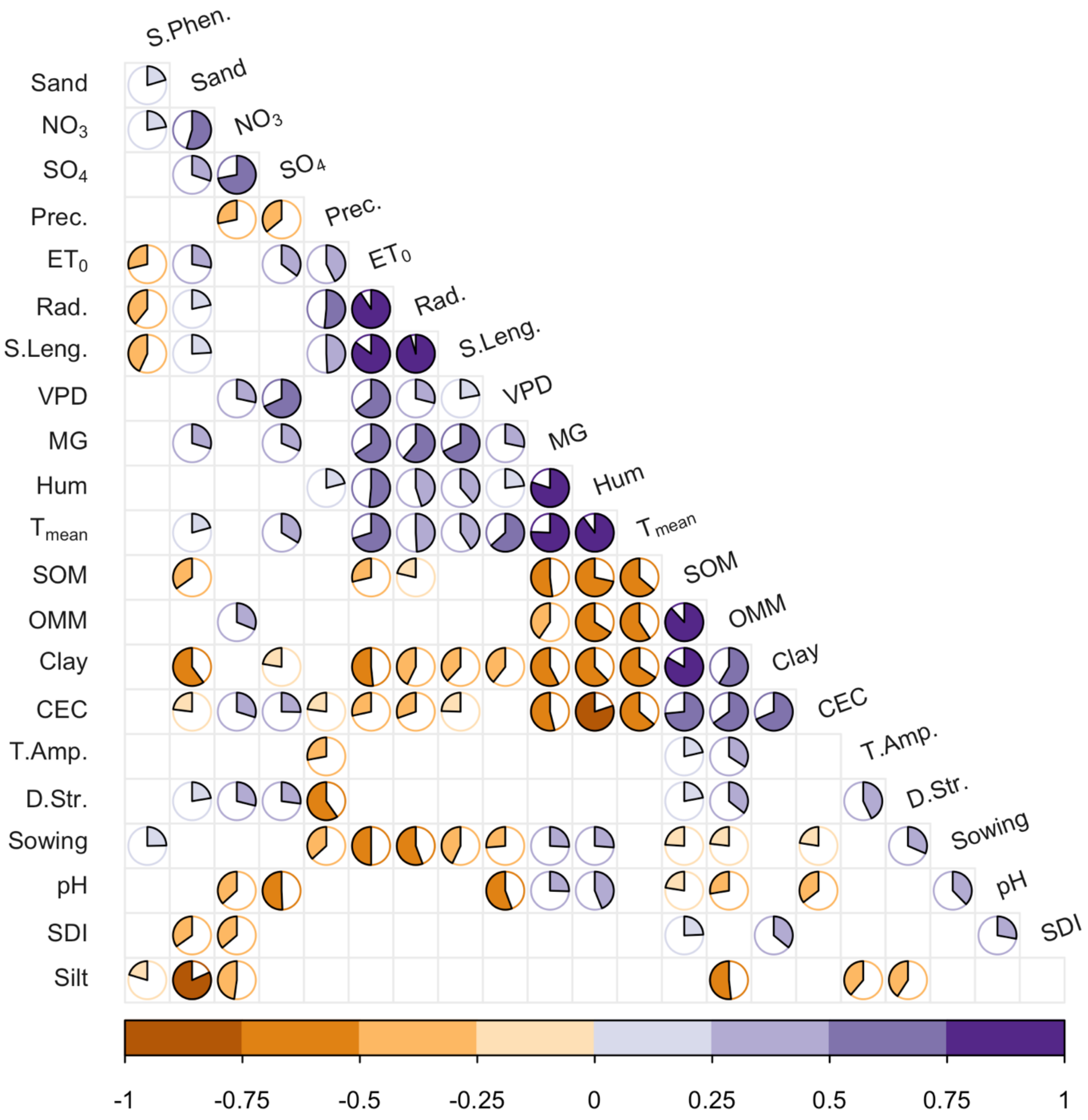

Supplementary Figure 2. The parsons' correlation matrix among weather and soil covariates. MG (maturity group); S.Phe (Ureide sampling stage); S.Length (season length); Sowing (sowing date); CEC (soil cation exchange capacity); Clay (clay relative content); N-NO3 (soil nitrate content); OMM (organic matter mineralization); pH (soil pH); Sand (sand relative content); Silt (silt relative content); S-SO4 (soil sulfate content); SOM (soil organic matter); ET0 (reference evapotranspiration); Hum (relative air humidity); Prec (rainfall precipitation); Rad (solar radiation); SDI (precipitation evenness); D.str (drought stress); T.Amp (air temperature amplitude); Tmean (air temperature); VPD (vapor pressure deficit).Weather covariates are presented for the entire crop

development, expressing overall association among covariates. The pie chart presence indicates a significant correlation at the level of p-value < 0.05.

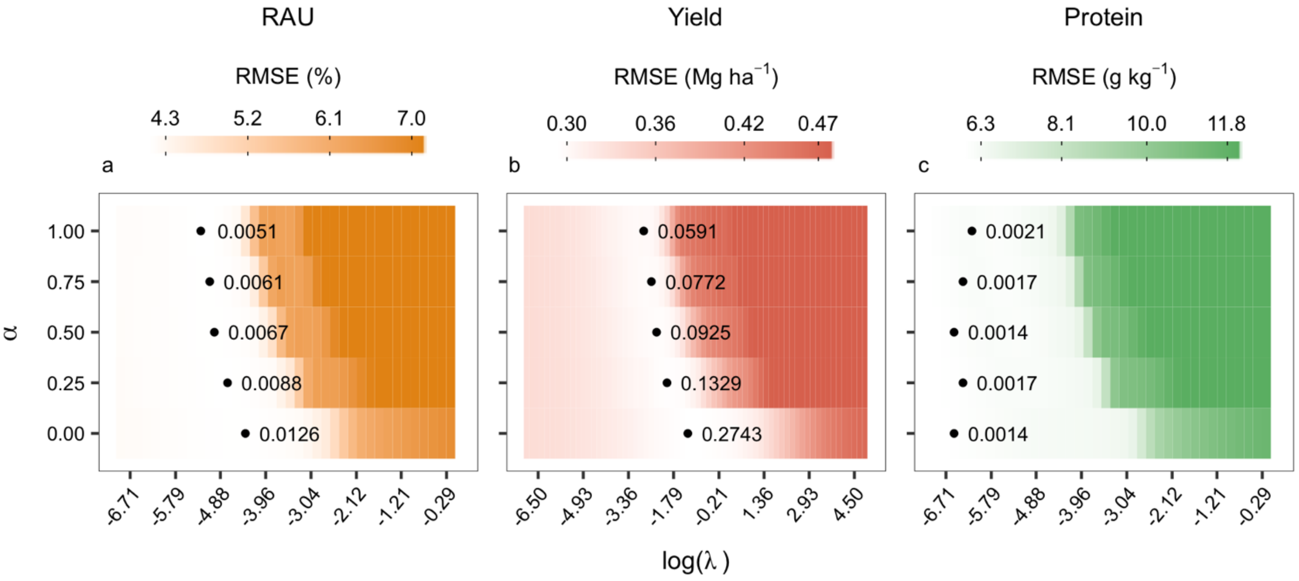

Supplementary Figure 3. Full model hyperparameter tuning for the relative abundance of the ureides (RAU), yield, and protein concentration. Dark colors represent high variance while light colors represent low variance. Black dots represent the lambda values minimizing the variance across all training-test sets.

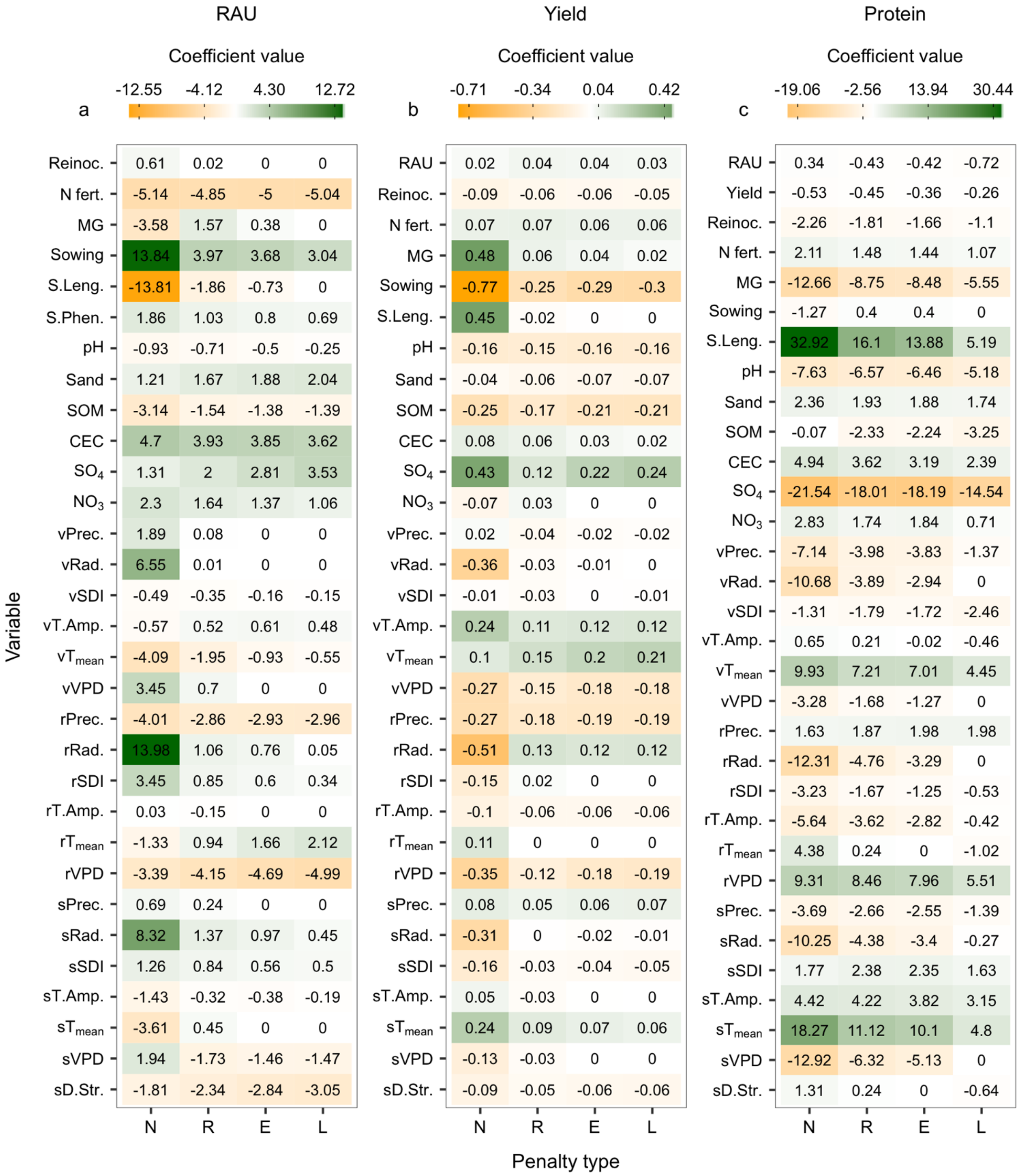

Supplementary Figure 4. Variables coefficient values for all parameters of the full model without penalization (N), ridge penalty (alpha = 0, [R]), elastic net (alpha=0.5 [E]), and LASSO penalization (alpha = 1 [L]). Green colors are the positive values whereas yellow colors are negative coefficient values.
